# The Role of Artificial Intelligence in Stroke Imaging in Emergency Settings: A Systematic Review

**DOI:** 10.7759/cureus.93941

**Published:** 2025-10-06

**Authors:** Anas E Ahmed, Wassal F Aljohani, Liyan K Abu Rukbah, Shahad A Rajhi, Norah K Najmi, Mohammed K Zughlul, Abdulrahman M Alshammari, Sultan D Alotaibi, Taghreed H Almarhabi, Mohammed A Al-Amri, Sama B Rebh

**Affiliations:** 1 Community Medicine, Jazan University, Jazan, SAU; 2 College of Medicine, Taif University, Taif, SAU; 3 College of Medicine, Jazan University, Jazan, SAU; 4 College of Medicine, Hail University, Hail, SAU; 5 College of Medicine, King Saud bin Abdulaziz University for Health Sciences, Riyadh, SAU; 6 College of Medicine, Umm Al-Qura University, Al-Qunfudhah, SAU; 7 College of Medicine, King Khalid University, Abha, SAU; 8 College of Medicine, Alexandria University, Alexandria, EGY

**Keywords:** alberta stroke program early ct score, artificial intelligence, clinical workflow, computed tomography, deep learning, emergency radiology, intracranial hemorrhage, large vessel occlusion, magnetic resonance imaging, stroke imaging

## Abstract

Rapid and accurate interpretation of neuroimaging is critical in acute stroke, but variability among human readers and the urgency of clinical workflows pose major challenges. Artificial intelligence (AI) has emerged as a promising adjunct in emergency stroke imaging, with the potential to enhance detection, scoring, and prognostication. We systematically reviewed the role of AI in this context, focusing on diagnostic performance, workflow feasibility, and implementation across key imaging modalities. A systematic search of PubMed, Scopus, Web of Science, and Cochrane CENTRAL was conducted from inception to August 20, 2025, following Preferred Reporting Items for Systematic reviews and Meta-Analyses (PRISMA) guidelines. Eligible studies were original English-language research that applied AI to emergency stroke imaging. Data were extracted on study design, population, imaging modality, AI architecture, performance metrics, workflow aspects, and interpretability, with study quality assessed using the Checklist for Artificial Intelligence in Medical Imaging (CLAIM). Nine studies met the inclusion criteria. AI models achieved high accuracy for intracranial hemorrhage (ICH) detection on non-contrast computed tomography (NCCT) scans, with area under the curve (AUC) values up to 0.98.

Real-world analyses reported balanced accuracy around 0.93 with near-real-time processing. Automated Alberta Stroke Program Early CT Score (ASPECTS) grading demonstrated almost perfect agreement with expert consensus (κ up to 0.90), outperforming individual radiologists in the hyperacute phase. Ischemic lesion detection using convolutional neural networks (CNNs) applied to magnetic resonance imaging (MRI) and computed tomography angiography (CTA) achieved accuracies of 83-86%. Symmetry-based methods further improved performance, though limitations were noted in posterior circulation strokes. Prognostic models integrating imaging and clinical data yielded moderate-to-good performance (AUC 0.79-0.91), with multimodal deep learning outperforming single-modality or clinical-only models. Workflow studies reported AI processing times of 2-4 minutes, although data transfer and system integration remained key bottlenecks. Interpretability tools, such as Gradient-weighted Class Activation Mapping (Grad-CAM) and Shapley Additive Explanations (SHAP), have enhanced transparency in several studies. Overall, AI demonstrates strong diagnostic and workflow potential in emergency stroke imaging, particularly for ICH detection, automated ASPECTS, and large vessel occlusion (LVO) alerts. Multimodal and transformer-based approaches show promise for outcome prediction and lesion segmentation, but further external validation and seamless integration into clinical workflows are required. AI is best positioned as a supportive tool to augment, rather than replace, clinical expertise in acute stroke care.

## Introduction and background

Stroke remains a leading cause of mortality and long-term disability worldwide, with acute ischemic stroke accounting for most cases. Timely diagnosis and treatment are critical, as each minute of delay causes progressive neuronal loss and worse functional outcomes [1]. The “golden hour” emphasizes that rapid recognition and intervention, particularly with reperfusion therapies, such as intravenous thrombolysis and endovascular thrombectomy, dramatically improve prognosis. Efficient emergency evaluation and rapid neuroimaging interpretation are therefore cornerstones of stroke care [2].

Neuroimaging is indispensable in acute stroke management. Non-contrast computed tomography (NCCT) is the first line to exclude intracranial hemorrhage and assess early ischemic changes, while computed tomography angiography (CTA) and computed tomography perfusion (CTP) provide information on large vessel occlusion and salvageable penumbral tissue [3]. Magnetic resonance imaging (MRI), particularly diffusion-weighted imaging (DWI), offers high sensitivity for early ischemia but is often limited in emergency settings [4]. Together, these modalities guide therapeutic decisions and patient triage but are constrained by time-sensitive interpretation, inter-observer variability, and resource limitations.

Traditional interpretation faces challenges that can directly impact outcomes. Subtle ischemic changes are frequently missed, Alberta Stroke Program Early CT Score (ASPECTS) interpretation shows high variability, and non-specialist centers often lack expert neuroradiologists [5]. These limitations can delay or misdirect treatment, emphasizing the need for technological support to augment human performance [6].

Artificial intelligence (AI), particularly machine learning and deep learning, is a promising solution in medical imaging. By learning patterns from large datasets, AI can detect subtle radiological features, standardize interpretation, and provide rapid results [7]. Applications in stroke imaging include hemorrhage detection on NCCT, automated ASPECTS scoring, large vessel occlusion detection on CTA, perfusion core and penumbra estimation, and functional outcome prediction [2].

Several studies show encouraging results. Automated ASPECTS tools achieve superior agreement with expert consensus, especially in the hyperacute phase [8]. AI algorithms show high sensitivity and specificity for intracranial hemorrhage detection, supporting triage in resource-limited settings. Multimodal models integrating MRI and clinical variables improve 90-day functional outcome prediction. Explainability tools, such as Gradient-weighted Class Activation Mapping (Grad-CAM) and Shapley Additive Explanations (SHAP), enhance trust by linking outputs to clinically interpretable features, though external validation and real-world integration remain limited [9].

Given the high stroke burden and the growing literature on AI applications, this review synthesizes evidence on AI models applied to NCCT, CTA, CTP, and MRI in hyperacute stroke, focusing on diagnostic accuracy, workflow integration, and prognostic utility, highlighting potential limitations and future directions.

## Review

Methods

Literature Search Strategy

This systematic review followed PRISMA guidelines [10]. A search was conducted in PubMed, Web of Science, Scopus, and the Cochrane Central Register of Controlled Trials (CENTRAL) from inception to August 20, 2025. Keywords included stroke, acute ischemic stroke, hemorrhagic stroke, cerebrovascular accident, brain infarction, artificial intelligence, machine learning, deep learning, neural network, convolutional neural network, computer-aided diagnosis, computed tomography, non-contrast CT, computed tomography angiography, perfusion imaging, Alberta Stroke Program Early CT Score, neuroimaging, emergency, hyperacute, and critical care. Boolean operators were applied, and search syntax was adapted according to the database. Only English-language human studies were included, with reference lists screened for a@dditional studies.

Eligibility Criteria

Eligibility followed the PICOS (Population, Intervention, Comparison, Outcomes, and Study) framework [11]. Studies were included if they: enrolled adult patients with suspected or confirmed acute ischemic or hemorrhagic stroke in emergency settings; applied AI, machine learning, or deep learning to CT, CTA, CTP, or MRI; compared AI performance with radiologists, established imaging standards, or clinical scoring systems; reported outcomes on diagnostic accuracy, lesion detection, ASPECTS scoring, intracranial hemorrhage, large vessel occlusion, perfusion assessment, or functional outcome prediction; and were original research. Exclusions included narrative reviews, editorials, conference abstracts without full text, animal studies, studies not focused on emergency stroke imaging, and non-English articles.

*Study Selection and Data Extraction*Records were imported into reference software, and duplicates were removed. Two reviewers independently screened titles and abstracts; full texts were reviewed for eligibility. Discrepancies were resolved by discussion or a third reviewer. A standardized extraction form captured study authors, country, design, sample size, population, imaging modality, clinical setting, reference standard, AI model details, performance outcomes (sensitivity, specificity, area under the curve, kappa), workflow aspects (processing time, integration, interpretability), and key findings. Extraction was performed independently by two reviewers with consensus for discrepancies.

Quality Assessment

Study quality was assessed using the Checklist for Artificial Intelligence in Medical Imaging (CLAIM), covering design, data handling, ground truth, model development, performance evaluation, explainability, and clinical integration [12,13]. Two reviewers performed assessments independently, resolving disagreements by consensus.

Results

Study Selection

**Figure 1:**
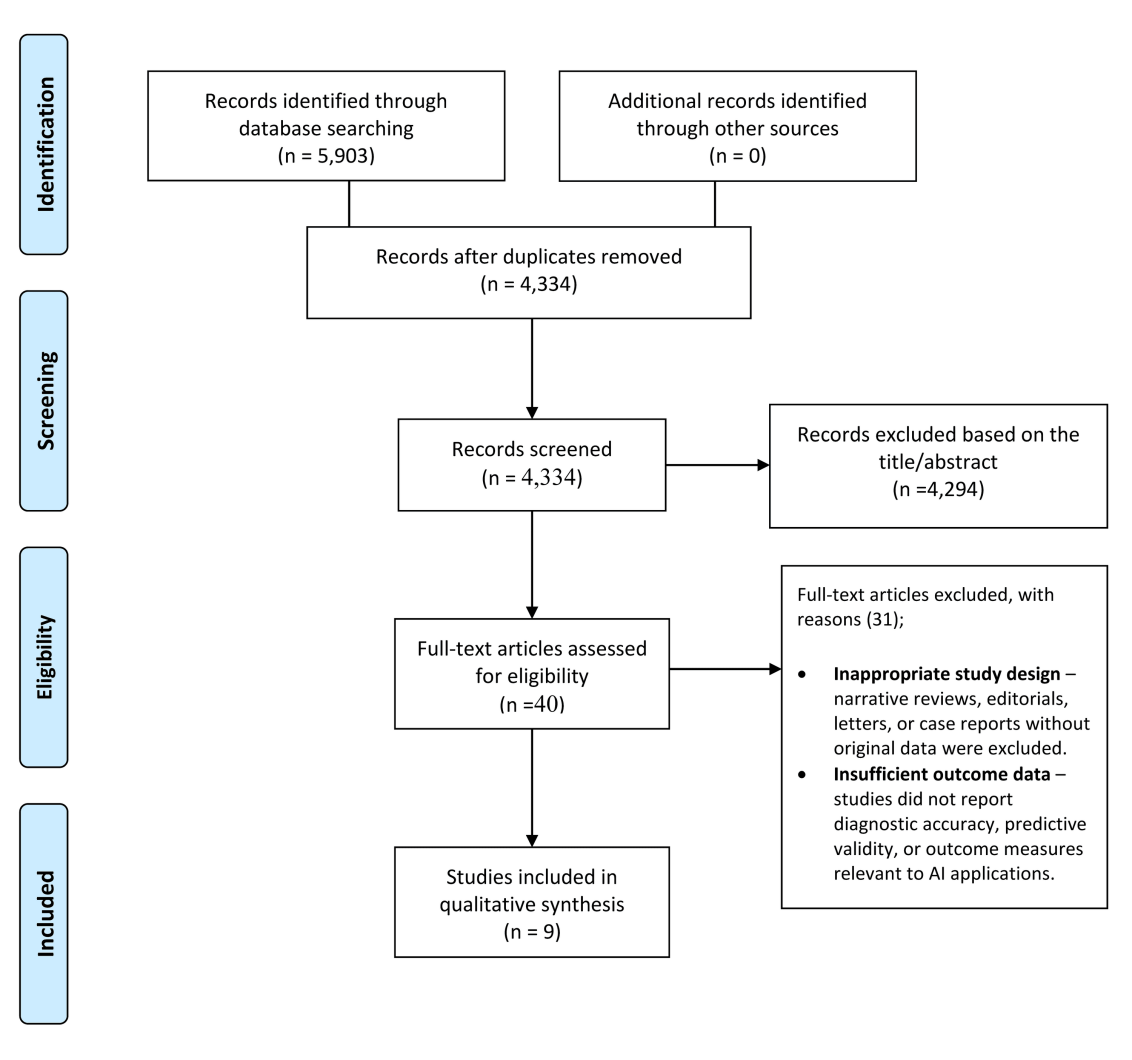
PRISMA flow diagram of the study selection process PRISMA: Preferred Reporting Items for Systematic Reviews and Meta-Analyses

From 5,903 records across PubMed, Cochrane Library, Scopus, and Web of Science, 4,334 unique records remained after duplicates were removed. Screening excluded 4,294, and 40 full texts were assessed, with 31 excluded for inappropriate design or insufficient outcome data. Nine studies were included in the qualitative synthesis (Figure 1) [2,9,13-19].

Study Characteristics

Studies applied AI to ischemic lesion detection, hemorrhage classification, automated ASPECTS scoring, and outcome prediction. Lee et al. applied CNNs to diffusion-weighted MRI, achieving 86.3% accuracy in anterior vs. posterior circulation classification [14]. Öman et al. trained 3D CNNs on CTA, showing contralateral hemisphere comparison improved sensitivity and Dice similarity, while adding NCCT offered little benefit (Table 1) [15].

**Table 1:** Characteristics, AI models, performance, and implementation in emergency stroke imaging studies This table summarizes the key characteristics, artificial intelligence (AI) model details, performance outcomes, and implementation aspects of studies applying AI to emergency stroke imaging. ACI = Acute cerebral ischemia, AIS = Acute ischemic stroke, ADC = Apparent diffusion coefficient, ANN = Artificial neural network, CBAM = Convolutional block attention module, CNN = Convolutional neural network, CT = Computed tomography, CTA = CT angiography, DNN = Deep neural network, DWI = Diffusion-weighted imaging, ED = Emergency department, FOV = Field of view, FLAIR = Fluid-attenuated inversion recovery, ICH = Intracranial hemorrhage, LR = Likelihood ratio, LVO = Large vessel occlusion, MCA = Middle cerebral artery, MRS = Modified Rankin Scale, ML = Machine learning, NCCT = Non-contrast CT, NIHSS = National Institutes of Health Stroke Scale, PCI = Posterior circulation infarct, RAPID = Rapid processing software for stroke imaging, RF = Random forest, SVM = Support vector machine, TOAST = Trial of Org 10172 in Acute Stroke Treatment, VCV = Cross-validation, V = Variable (used in models), ASTRAL = Acute Stroke. Treatment Registry and Analysis of Lausanne

Study	Country	Study Design	Sample Size & Population	Imaging Modality	Clinical Setting	Comparator	AI Model Characteristics	Performance Outcomes	Implementation Aspects	Findings
Dawud et al. [2]	Cyprus/Nigeria	Experimental, deep learning with transfer learning	12,635 CT images (8,855 training, 3,790 testing; normal + hemorrhage)	Non-contrast CT (NCCT)	Emergency neuroradiology (ICH detection)	No radiologist comparator	CNN from scratch; pretrained AlexNet; AlexNet-SVM; transfer learning applied	CNN: 90.65%; AlexNet: 92.13%; AlexNet-SVM: 93.48%; Sensitivity up to 95%, specificity up to 90%; MSE lowest with AlexNet-SVM (0.054)	MATLAB; Intel i7 CPU, 16GB RAM; transfer learning reduced training; data augmentation applied	Transfer learning improved performance; AlexNet-SVM highest accuracy and generalization. Feasible for brain hemorrhage detection in emergency CT.
Abedi et al. [9]	USA & Greece	Prospective registry-based pilot	260 patients (130 acute cerebral ischemia, 130 stroke mimics); mean age 57±15, 48% male; ≤4.5 h from onset	Clinical + imaging/lab data (CT, MRI)	EDs of two tertiary stroke centers	Final diagnosis by clinical course, CT/MRI, MRA/CTA	ANN using backpropagation; trained in R (Neuralnet); 10-fold cross-validation; optimized hidden neurons (6 units)	Sensitivity 80% (95% CI 71.8–86.3); Specificity 86.2% (95% CI 78.7–91.4); Median precision for ACI 92%; Positive LR 5.8	ANN trained on balanced datasets; compared with logistic regression; validated with 10-fold CV	ANN distinguished ACI from stroke mimics with good accuracy; outperformed logistic regression; potential rapid ED screening tool; needs larger validation.
Jung et al. [13]	South Korea	Prospective multicenter registry; retrospective ML analysis	2,606 AIS patients; 993 (38.1%) poor outcome (mRS 3–6)	MRI: DWI (b1000, ADC), FLAIR + clinical metadata (22 variables)	Multicenter tertiary stroke centers	Compared against single-modality models	Ensemble DL: 3D CNNs for MRI (ResNeXt+CBAM) + fully connected DNN for clinical data; probability fusion	AUC 0.830 (CV), 0.779 (time-based CV); Sensitivity 0.759, Specificity 0.743, F1=0.696	Python (TensorFlow 2.9, Scikit-learn 1.1.3); Linux workstation (i9, dual RTX 2080Ti, 64GB RAM); SHAP + Grad-CAM	Multimodal ensemble outperformed single-modality models; age & NIHSS most influential; imaging attention maps localized lesions; robust across 18 centers; limited to MRI-available settings.
Lee et al. [14]	Taiwan	Retrospective, single-center	127 patients (42–98 yrs; mean 70.2; 60M/67F) with hyperacute ischemic stroke (0–24 h)	MRI – Diffusion-Weighted Imaging (DWI)	Emergency; suspected hyperacute infarct	Ground-truth by 2 radiologists (consensus)	Three CNN models: Inception-v3, EfficientNet-b0, modified LeNet; trained with transfer learning, 7:3 validation split	Inception-v3: accuracy 86.3%, F1 86.2%, κ=0.715; Modified LeNet: accuracy 85.2%, F1 84.7%, κ=0.693; EfficientNet-b0: accuracy 83.6%, F1 83.0%, κ=0.662. Best sensitivity for normal images, poorest for PCI (42–49%)	MATLAB 2021b; Intel i7 CPU, 16GB RAM; Grad-CAM for interpretability; training time 35–410 min	CNNs detected hyperacute ischemic stroke and classified vascular territory (ACI vs PCI). Inception-v3 performed best; misclassifications due to artifacts. Supports aid in emergency stroke triage.
Öman et al. [15]	Finland	Retrospective, single-center feasibility	60 patients with suspected acute ischemic stroke (30 stroke-positive MCA/ICA occlusion, 30 controls); median age 73	CT Angiography (CTA source images); NCCT included in some models	Emergency stroke protocol	Manual lesion segmentation by neuroradiologist + radiologist (consensus)	3D CNN (DeepMedic) trained on CTA alone, CTA+hemispheric comparison, CTA+hemispheric comparison+NCCT; 30/30 train/test split	Sensitivity: 0.67 (CTA), 0.74 (CTA+hemi), 0.71 (CTA+hemi+NCCT); Specificity: 0.93–0.96; ROC-AUC: 0.91–0.93; Dice up to 0.61	DeepMedic (Theano-based); training 77–152 h; per-patient ~3–4 min; hemispheric comparison improved specificity; NCCT minor added value	3D CNN accurately detected and lateralized acute ischemic stroke. Hemispheric comparison reduced false positives; small lesions overestimated, large lesions underestimated. Feasible for rapid CTA-based stroke detection.
Heo et al. [16]	South Korea	Retrospective cohort using a prospective registry	2,604 AIS patients (≤7 days from onset; excluded prestroke mRS>2 or recanalization therapy); mean age 66.2 ± 12.6, 61.7% male	Clinical + baseline imaging (stroke classification, NIHSS, labs, etc.)	Tertiary stroke center registry	ASTRAL prognostic score	ML: Deep Neural Network (3 hidden layers, 15 units), Random Forest (300 trees), Logistic Regression; compared with ASTRAL	mRS 0–2 at 3 months: DNN AUC 0.888 (vs ASTRAL 0.839, p<0.001); RF AUC 0.857; LR AUC 0.849	TensorFlow v1.1, Scikit-learn v0.18; 67/33% train/test split; stats in R	DNN improved long-term outcome prediction; RF and LR similar to ASTRAL. Suggests ML (especially DNN) can enhance prognostic accuracy.
Kuang et al. [17]	Canada & South Korea	Single-center prospective registry; ML development & validation	257 AIS patients (<8 h onset); NCCT + DWI within 1 h; 157 training, 100 testing; median age 69–70	NCCT (5 mm slices) with DWI reference	AIS patients within 8 h	Expert ASPECTS readings on DWI	ML: Random Forest + 376 texture features; region-level classification	Total ASPECTS ICC=0.76; region κ=0.60; Dichotomized ASPECTS (≤4 vs >4): κ=0.78; Sensitivity 97.8%, Specificity 80%, AUC 0.89; Regional AUC up to 0.81	Python/Scikit-learn; ~400 texture features per ASPECTS region; median NCCT-DWI gap ~39 min	ML-based ASPECTS had strong agreement with DWI-ASPECTS; superior to expert NCCT-ASPECTS; high sensitivity supports triage for reperfusion therapy; needs external validation.
Maegerlein et al. [18]	Germany	Retrospective, single-center	152 patients: Cohort 1: 100 MCA occlusion thrombectomy, mean 73 ±14; Cohort 2: 52 suspected stroke, no LVO, mean 73 ±16	Non-contrast CT (NCCT)	Acute stroke candidates for thrombectomy / suspected stroke	Consensus ASPECTS by 2 neuroradiologists (baseline CT + follow-up MRI)	Automated RAPID ASPECTS (ML-based classification of ASPECTS regions)	Software k=0.90; neuroradiologists k=0.56–0.57; 1–4 h: software k=0.78 vs readers k=0.27–0.36; >4 h: k=0.76–0.92; dichotomized ASPECTS ≥6: software k=0.70 vs readers 0.40–0.50	Processing 2–4 min; RAPID platform; ~20% scans limited by FOV/quality; requires review	Automated software outperformed neuroradiologists for early infarct detection; improved reliability for thrombectomy selection.
van Os et al. [19]	Netherlands	Observational registry (MR CLEAN, 18 centers)	1,383 EVT patients with anterior circulation LVO; mean age 69.8, 54% male	Baseline NCCT, CTA, DSA + treatment data	EVT centers	Logistic regression with variable selection (backward elimination, LASSO, Elastic Net)	ML: Random Forest, SVM, ANN, Super Learner ensemble; compared with logistic regression	Reperfusion prediction: poor (AUC 0.53–0.57). 3-month mRS ≤2: moderate (AUC 0.77–0.79); with baseline+treatment data: good (AUC 0.88–0.91); no ML advantage over LR	Nested CV (100 random splits, 10-fold inner CV); Scikit-learn; code public	ML did not outperform logistic regression; good discrimination for functional outcome when treatment included; limited improvement for reperfusion prediction.

The use of AI improved emergency triage. Abed et al. distinguished ischemic stroke from mimics with 80% sensitivity and 86% specificity, outperforming logistic regression [9]. Dawud et al. achieved 93.5% accuracy in intracranial hemorrhage detection using transfer learning with AlexNet-SVM [2]. Prognostic models integrated MRI and clinical data, outperforming single-modality models [13,16]. Automated ASPECTS scoring demonstrated higher agreement with reference standards than individual neuroradiologists, with 2-4-minute processing times [17,18].

Quality Assessment

The CLAIM-based appraisal revealed variability in methodological rigor and transparency [13]. Most studies provided clear dataset descriptions and appropriate ground truth references (radiologist consensus, diffusion-weighted imaging, or adjudicated outcomes) [9,13-19]. Some studies were prospective registry-based, while others were retrospective or unclear [2,9,13,17,19]. Train/validation/test separation was generally adequate, but external validation was limited. Common weaknesses included absent sample size justification, insufficient reporting of missing data handling, and limited details on model architecture and hyperparameters. Performance metrics were typically appropriate, but calibration and threshold analyses were rarely reported. Explainability methods were applied in only a few studies (Grad-CAM, SHAP, and Grad-CAM [13,14]), and descriptions of clinical workflow integration were inconsistent. No studies provided code or data availability. Ethics approval was stated in nearly all cases, but the lack of external validation and reproducibility remains a significant limitation (Table 2).

**Table 2:** Quality assessment of included studies on AI applications in acute stroke imaging Y = Yes, N = No, P = Partially, U = Unclear, CV = Cross-validation, DWI = Diffusion-weighted imaging, FLAIR = Fluid-attenuated inversion recovery, mRS = modified Rankin Scale, EVT = Endovascular thrombectomy, FU = follow-up, ASPECTS = Alberta Stroke Program Early CT Score, ML = Machine learning, SHAP = Shapley additive explanations, Grad-CAM = Gradient-weighted class activation mapping, NCCT = Non-contrast computed tomography, CTA = Computed tomography angiography, MRI = Magnetic resonance imaging

Study (Authors [Ref])	Prospective design reported	Dataset description clear (source/timeframe)	Inclusion/Exclusion stated	Ground truth appropriate	Clear train/val/test separation	External validation	Sample size justification	Missing data handling	Model details reproducible (arch/hparams)	Appropriate metrics (with CIs)	Calibration/thresholds reported	Explainability used	Clinical workflow/integration described	Code/Data availability	Ethics/approval stated
Dawud [2]	N	P	U	U	Y	N	N	U	P	P	N	N	U	N	U
Abedi [9]	P (registry)	Y	Y	Y (adjudicated Dx)	Y (CV)	N	N	U	P	Y	N	N	P	N	Y
Jung [13]	P (prospective registry; retrospective ML)	Y	Y	Y (DWI/FLAIR + clinical)	Y (CV incl. time-based)	N (multicenter internal)	N	P	P	Y	P	Y (SHAP/Grad-CAM)	Y	N	Y
Lee [14]	N	Y	P	Y	Y	N	N	U	P	P	N	Y	P	N	Y
Öman [15]	N	Y	Y	Y (consensus seg.)	Y	N	N	U	P	P	N	N	P	N	Y
Heo [16]	P (registry)	Y	Y	Y (mRS 90d)	Y (67/33)	N	N	U	P	Y	N	N	P	N	Y
Kuang [17]	P (registry)	Y	Y	Y (DWI ASPECTS)	Y (hold-out)	N	N	U	P	Y	P	N	P	N	Y
Maegerlein [18]	N	Y	Y	Y (CT+FU MRI consensus)	Y	N	N	U	P	P	N	N	Y (RAPID use/time)	N	Y
van Os [19]	P (registry)	Y	Y	Y (mRS/EVT data)	Y (nested CV)	N (multicenter internal)	N	P	P	Y	P	N	P	N	Y

Intracranial Hemorrhage Detection on NCCT

AI models demonstrated strong potential in automated intracranial hemorrhage detection on NCCT in emergency settings. Dawud et al. compared CNNs trained from scratch, pretrained AlexNet, and AlexNet-SVM on 12,635 CT images [2]. Transfer learning with AlexNet-SVM achieved the highest accuracy (93.5%) and the lowest mean squared error, enabling near-real-time triage compatible with acute workflows.

Ischemic Lesion Detection and Vascular Territory Classification

Lee et al. applied Inception-v3, EfficientNet-b0, and modified LeNet CNNs to diffusion-weighted MRI from 127 hyperacute ischemic stroke patients [14]. Inception-v3 achieved 86.3% validation accuracy and moderate kappa agreement (0.715), classifying anterior versus posterior circulation strokes, though posterior misclassifications persisted. Grad-CAM highlighted lesion regions, improving interpretability. Öman et al. trained a 3D CNN (DeepMedic) on CTA images [15]. Contralateral hemisphere comparison improved sensitivity (0.74 vs. 0.67) and Dice similarity (0.55 vs. 0.40), while adding NCCT offered no additional benefit. False positives were often linked to periventricular white matter or cortical sulci, reflecting age-related changes.

Automated ASPECTS Scoring on NCCT

Kuang et al. developed a random forest-based approach using NCCT textural features in 257 patients within eight hours of onset, using diffusion-weighted imaging ASPECTS as reference [17]. The model achieved an intraclass correlation of 0.76, strong dichotomized scoring (≤4 vs. >4: κ = 0.78, sensitivity 97.8%, specificity 80%, AUC 0.89), outperforming expert CT-ASPECTS. Maegerlein et al. validated commercial RAPID ASPECTS software in 152 patients, showing almost perfect agreement with consensus scoring (κ = 0.90) and processing times of 2-4 minutes, supporting acute workflow integration [18].

Functional Outcome Prediction (90-Day Modified Rankin Scale)

Heo et al. compared machine learning models to the ASTRAL score in 2,604 patients, finding that deep neural networks achieved higher AUC (0.888 vs. 0.839) [16]. Van Os et al. found machine learning and logistic regression performed similarly for reperfusion prediction (AUC 0.53-0.57) but moderately to well for functional outcomes (AUC 0.77-0.91) [19]. Jung et al. applied a multimodal deep learning ensemble combining MRI sequences and clinical data in 2,606 patients, achieving superior AUCs (0.83 standard cross-validation, 0.779 time-based) and improved interpretability with SHAP and Grad-CAM [13].

Stroke Versus Stroke Mimics in Emergency Department Triage

Abed et al. trained an artificial neural network on 260 patients (130 acute ischemic strokes, 130 stroke mimics), achieving 80% sensitivity and 86.2% specificity, outperforming logistic regression [9]. Missing data limited generalizability, highlighting the need for robust validation in diverse populations.

Workflow and Implementation

Processing times for AI tools were compatible with acute stroke workflows. RAPID ASPECTS generated results in two to four minutes, and Öman et al. reported CTA-based CNN processing of three to four minutes per patient [15,18]. Explainability methods, including Grad-CAM and SHAP, improved clinician trust [13,14]. Challenges affecting robustness included temporal sensitivity (>270 minutes from onset to NCCT), aging-related artifacts, and limited field-of-view, emphasizing the need for human oversight in deployment [15,17,18].

## Conclusions

This systematic review demonstrates that AI has strong potential to enhance emergency stroke imaging by improving diagnostic accuracy, reproducibility, and workflow efficiency. The most clinically mature applications include intracranial hemorrhage detection on NCCT, automated ASPECTS scoring, and proximal large-vessel occlusion alerts, all of which can support rapid triage and treatment selection. Emerging evidence also supports the use of multimodal models for ischemic lesion segmentation and functional outcome prediction, although their added value over optimized clinical tools remains modest and requires further external validation. Workflow considerations, including data transfer bottlenecks, interpretability, and robustness across imaging protocols, remain key barriers to implementation. Overall, AI represents a promising adjunct to neuroradiologists in acute stroke care, but widespread adoption will depend on rigorous multicenter validation, standardized reporting, and seamless integration into emergency workflows.
